# Association between the *IL1B (-511)*, *IL1B (+3954)*, *IL1RN (VNTR)* Polymorphisms and Graves' Disease Risk: A Meta-Analysis of 11 Case-Control Studies

**DOI:** 10.1371/journal.pone.0086077

**Published:** 2014-01-21

**Authors:** Min-Li Chen, Ning Liao, Hua Zhao, Jian Huang, Zheng-Fu Xie

**Affiliations:** 1 Department of Geriatrics and Gerontology, First Affiliated Hospital, Guangxi Medical University, Nanning, China; 2 Department of Clinical Medicine, Grade 2001, Guangxi Medical University, Nanning, China; IPATIMUP/Faculty of Medicine of the University of Porto, Portugal

## Abstract

**Background:**

Data on the association between the interleukin-1 (*IL-1*) gene polymorphisms and Graves' disease (GD) risk were conflicting. A meta-analysis was undertaken to assess this association.

**Methods:**

We searched for case-control studies investigating the association between the *IL1B* (*-511*), *IL1B* (*+3954*), *IL1RN (VNTR)* polymorphisms and GD risk. We extracted data using standardized forms and calculated odds ratios (OR) with 95% confidence intervals (CI).

**Results:**

A total of 11 case-control studies were included in this meta-analysis. Available data indicated that the *IL1B* (*-511*) polymorphism was associated with GD risk in the overall populations (Caucasians and Asians) in homozygote model (TT vs. CC, OR = 0.86, 95% CI: 0.76–0.97, *P_z_* = 0.015), but not in dominant and recessive models (TT+TC vs. CC: OR = 0.95, 95% CI: 0.81–1.12, *P_z_* = 0.553 and TT vs. TC+CC: OR = 0.82, 95% CI: 0.60–1.12, *P_z_* = 0.205, respectively). No association between the *IL1B* (*+3954*), *IL1RN (VNTR)* polymorphisms and GD risk was found in the overall populations in any of the genetic models. In subgroup analyses according to ethnicity, the *IL1B* (*-511*) polymorphism was associated with GD risk in Asians in recessive and homozygote models (TT vs. TC+CC: OR = 0.68, 95% CI: 0.55–0.84, *P_z_*<0.001 and TT vs. CC: OR = 0.81, 95% CI: 0.70–0.93, *P_z_* = 0.003, respectively), but not in dominant model (TT+TC vs. CC: OR = 0.92, 95% CI: 0.77–1.11, *P_z_* = 0.389). No association between the *IL1B* (*+3954*), *IL1RN (VNTR)* polymorphisms and GD risk was indicated in Asians, and we found no association between the *IL1B* (*-511*), *IL1B* (*+3954*), *IL1RN (VNTR)* polymorphisms and GD risk in Caucasians in any of the genetic models.

**Conclusion:**

The *IL1B* (*-511*) polymorphism, but not the *IL1B* (*+3954*) and *IL1RN (VNTR)* polymorphisms was associated with GD risk in Asians. There was no association between these polymorphisms and GD risk in Caucasians.

## Introduction

Graves' disease (GD) is an autoimmune thyroid disorder characterized by hyperthyroidism. It is commonly seen in young and middle-aged people, affecting 0.5–1.0% of the general population [1]. In GD, circulating autoantibodies are produced, which target well-defined thyroidal antigens, such as thyroid peroxidase, thyroglobulin, and the thyroid-stimulating hormone receptor (TSHR) [2]. Stimulatory autoantibodies binding to TSHR on thyroid follicular cells induce over-synthesis of the thyroid hormones thyroxine (T4) and triiodothyronine (T3). The systematic effects of elevated T3/T4 manifests as a sped up metabolism and its correlated symptoms. In addition to hyperthyroidism, some patients with GD have clinical involvement of the eyes resulting in Graves' Ophthalmopathy (GO) and the skin resulting in localized myxoedema [3], [4].

The etiology of GD remains unknown; however, it is considered to be caused by an interplay between genetic and environmental factors [5], [6]. In common with other autoimmune diseases, GD has a complex genetic basis, with numerous different genes each contributing in various degrees to the inherited susceptibility. Using both the candidate gene approach and whole genome linkage studies, some major susceptibility genes to GD have been identified, including human leukocyte antigen (HLA) on chromosome 6p21, *Cytotoxic T-Lymphocyte Antigen (CTLA) -4* on chromosome 2q33 and *lymphoid tyrosine phosphatase (LYP)* on chromosome 1p13 [7]–[10]. Besides these, it is believed that additional genes contribute to the genetic susceptibility to GD, as well as to its different phenotypes. Much interest has currently centered around the exploration of additional susceptibility genes to GD, which may help elucidate signaling pathways involved in the pathogenesis of GD, and help develop novel therapeutic strategies for the disease.

The interleukin-1 (IL-1) family is a group of related cytokines including two agonist proteins (IL-1A and IL-1B) and an antagonist protein (IL-1RA). The genes encoding for these molecules are located close to one another on chromosome 2q13-14, which are candidate genes for GD. There are several common polymorphisms that have been most frequently investigated. The *IL1B* gene has two single nucleotide polymorphisms (SNP) at position -511 in the promoter region (rs16944) and at position +3954 in the fifth exon (rs1143634), respectively [11], [12]. In the *IL1RN* gene, the second intron contains a variable number of tandem repeats (VNTR) of 86 base pairs (rs2234663) [13]. Many genetic association studies have intensively investigated the association of these SNPs with GD risk; however, results from individual studies are not consistent [14]–[27]. Here we conducted a meta-analysis with pooled data from 11 genetic association studies to determine risk of GD associated with these three polymorphisms in the *IL-1* gene.

## Materials and Methods

### Search strategy and selection of studies

We conducted a literature search for studies reporting on the association between *IL-1* gene polymorphisms and GD risk using the electronic database PubMed, Medline and Web of Science from January 1990 up to the end of July 2013. The search results were limited to publications in English. The search strategy used the key words “IL-1, Graves' disease, thyroid disease, genetic, association, polymorphism, susceptibility”. We also screened references of retrieved publications. Inclusion criteria were as follows: (a) case-control design; (b) sufficient data provided to reconstruct two by two tables or determine odds ratio and confidence intervals. Studies were excluded if one of the following existed: (a) no control; (b) no usable data reported.

### Data extraction

Information on general study characteristics including first author, year of publication, country, ethnicity, sample size, and genotype distribution in cases and controls was collected from the published papers.

### Statistical analyses

Data were analyzed using Stata 11.0. We used raw data of genotype distribution, without adjustment for calculation of the study-specific estimates of odds ratio (OR) and 95% confidence interval (CI). Dominant, recessive and homozygote genetic models were employed to assess the relation of *IL-1* gene polymorphisms with GD risk. Z-test was conducted for assessing the significance of the pooled ORs, with p<0.05 considered statistically significant. Cochran' s Q test was used to test for heterogeneity, with significance level set at 0.10. The pooled estimation of the ORs of each publication was calculated with the fixed effects model (Mantel-Haenszel methods) in the absence of significant between-study heterogeneity [28], while the random effects model (DerSimonian and Laird' s method) was employed for results showing high heterogeneity [29]. We used Begg' s test to evaluate publication bias.

## Results

### Study characteristics

**Figure 1:**
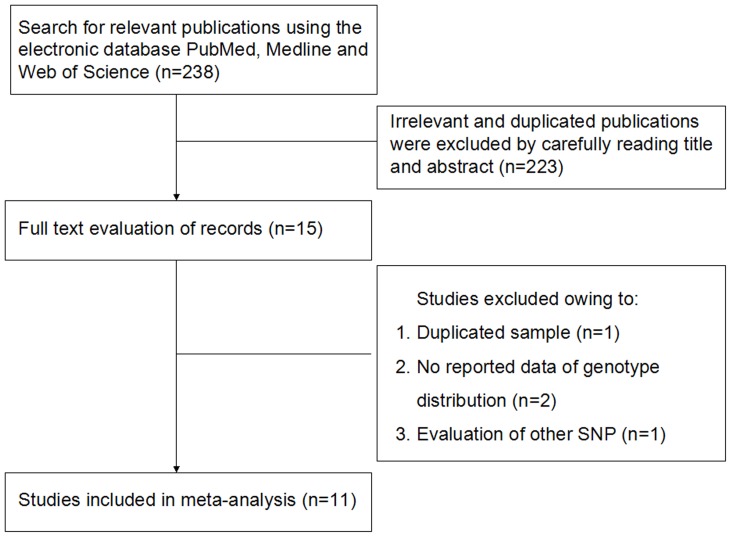
Flowchart of study selection process.

Figure 1 summarized the process of identifying eligible studies. After title and abstract evaluation we were left with 15 studies. Four studies were excluded after evaluating the remaining 15 articles in their entirety. Finally, 11 studies were included in the meta-analysis [14]–[16], [19]–[25], [27]. Table 1 summarizes the characteristics of the 11 studies according to the *IL-1* gene polymorphisms. Six studies investigated the *IL1B* (*-511*) polymorphism [20], [22]–[25], [27], six the *IL1B* (*+3954*) polymorphism [19], [20], [22]–[24], [27] and six studies the *IL1RN* (*VNTR*) polymorphism [14]–[16], [20]–[22]. Table 2 summarizes genotype distribution of the *IL-1* gene polymorphisms.

**Table 1:** Characteristics of the studies evaluating *IL-1* gene polymorphisms and GD risk.

First author	Year	Country or Area	Ethnicity	Patients (n)	Controls (n)	Polymorphism
Blakemore et al. [14]	1995	UK	Caucasians	100	261	*IL1RN (VNTR)*
Cuddihy et al. [15]	1996	USA	Caucasians	141	145	*IL1A* (*+4845*) and *IL1RN* (*VNTR*)
Mühlberg et al. [16]	1998	Germany	Caucasians	144	174	*IL1RN (VNTR)*
Reichmann et al. [19]	2004	Germany	Caucasians	53	259	*IL1B* (*+3954*)
Chen et al. [20]	2005	Taiwan	Asians	95	163	*IL1B (-511)* and *(+3954)* and *IL1RN (VNTR)*
Nakkuntod et al. [21]	2006	Thailand	Asians	137	137	*IL1RN* (*VNTR*)
Kammoun-Krichen et al. [22]	2007	Tunisia	Caucasians	131	225	*IL1A* (*-889*), *IL1B (-511)* and *(+3954)* and *IL1RN* (*VNTR*)
Lacka et al. [23]	2009	Poland	Caucasians	117	106	*IL1B (-511)* and *(+3954)*
Khalilzadeh et al. [24]	2009	Iran	Caucasians	107	140	*IL1A (-889), IL1B (-511) and (+3954), IL1RN (Mspa-1 11100)*
Liu et al. [25]	2010	China	Asians	760	735	*IL1A* (*-889*) and *IL1B (-511)*
Liu et al. [27]	2010	Taiwan	Asians	484	160	*IL1B (-31), (-511)* and *(+3954)*

IL-1, interleukin-1; UK, united kingdom; USA, united states of America.

**Table 2:** Genotype distribution of *IL-1* polymorphisms in patients and controls.

Polymorphism	Patients				Controls			
*IL1B* (*-511*)	CC (%)	TC (%)	TT (%)	Total	CC (%)	TC (%)	TT (%)	Total
Chen (2005)	29 (30.53)	49 (51.58)	17 (17.89)	95	34 (20.86)	78 (47.85)	51 (31.29)	163
Kammoun-Krichen (2007)	17 (12.98)	86 (65.65)	28 (21.37)	131	26 (11.56)	165 (73.33)	34 (15.11)	225
Lacka (2009)	61 (52.14)	40 (34.19)	16 (13.68)	117	67 (63.21)	28 (26.42)	11 (10.38)	106
Khalilzadeh (2009)	33 (30.84)	60 (56.07)	14 (13.08)	107	36 (25.90)	82 (58.99)	21 (15.11)	139
Liu (2010)	228 (30.00)	399 (52.50)	133 (17.50)	760	220 (29.93)	351 (47.76)	164 (22.31)	735
Liu (2010)	154 (32.70)	230 (48.83)	87 (18.47)	471	48 (30.00)	70 (43.75)	42 (26.25)	160
*IL1B* (*+3954*)	CC (%)	TC (%)	TT (%)	Total	CC (%)	TC (%)	TT (%)	Total
Reichmann (2004)	3 (5.67)	26 (49.06)	24 (45.28)	53	14 (5.41)	101 (39.00)	144 (55.60)	259
Chen (2005)	90 (94.74)	5 (5.26)	0 (0.00)	95	155 (95.09)	7 (4.29)	1 (0.61)	163
Kammoun-Krichen (2007)	26 (19.85)	92 (70.23)	13 (9.92)	131	47 (20.89)	155 (68.89)	23 (10.22)	225
Lacka (2009)	77 (65.81)	32 (27.35)	8 (6.84)	117	78 (73.58)	24 (22.64)	4 (3.77)	106
Khalilzadeh (2009)	48 (45.71)	51 (48.57)	6 (5.71)	105	70 (50.00)	58 (41.43)	12 (8.57)	140
Liu (2010)	458 (97.24)	13 (0.03)	0 (0.00)	471	36 (22.50)	80 (50.00)	44 (27.5)	160
*IL1RN* (*VNTR*)	LL (%)	2L (%)	22 (%)	Total	LL (%)	2L (%)	22 (%)	Total
Blakemore (1995)	45 (45.00)	42 (42.00)	13 (13.00)	100	152 (58.24)	92 (35.25)	17 (6.51)	261
Cuddihy (1996)	83 (58.87)	50 (35.46)	8 (5.67)	141	83 (57.24)	51 (35.17)	11 (7.59)	145
Mühlberg (1998)	99 (68.75)	33 (22.92)	12 (8.33)	144	104 (59.77)	49 (28.16)	21 (12.07)	174
Chen (2005)	87 (91.58)	7 (7.37)	1 (1.05)	95	147 (90.18)	15 (9.20)	1 (0.61)	163
Nakkuntod (2006)	109 (79.56)	25 (18.25)	3 (2.19)	137	106 (77.37)	29 (21.17)	2 (1.46)	137
Kammoun-Krichen (2007)	92 (70.23)	35 (26.72)	4 (3.05)	131	158 (70.22)	63 (28.00)	4 (1.78)	225

IL-1, interleukin-1.

### Association between the *IL1B* (*-511*) polymorphism and GD

**Figure 2:**
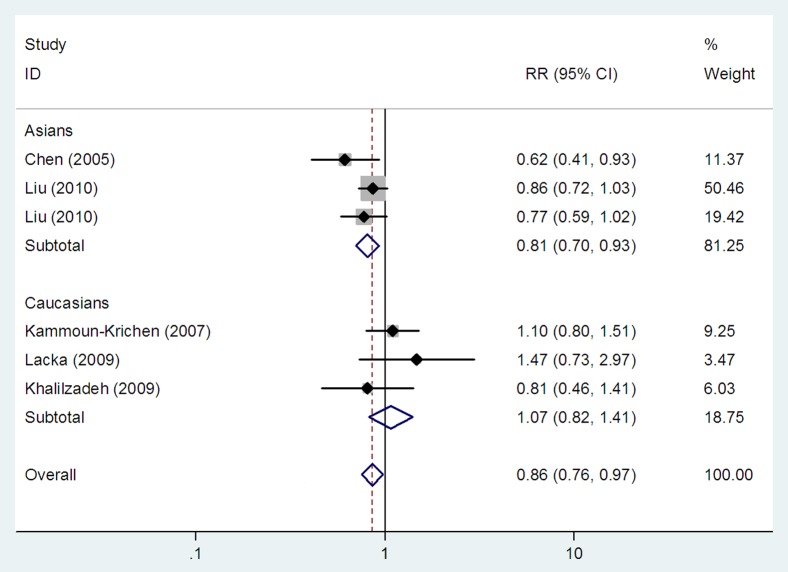
Meta-analysis with a fixed effects model for the association between the *IL1B* (*-511*) polymorphism and GD risk in homozygote model. Each study is shown by the point estimate of the odds ratio, and a horizontal line denotes the 95% confidence interval. The pooled odds ratio is represented by a diamond. The area of the grey squares reflects the weight of the study in the meta-analysis.

**Figure 3:**
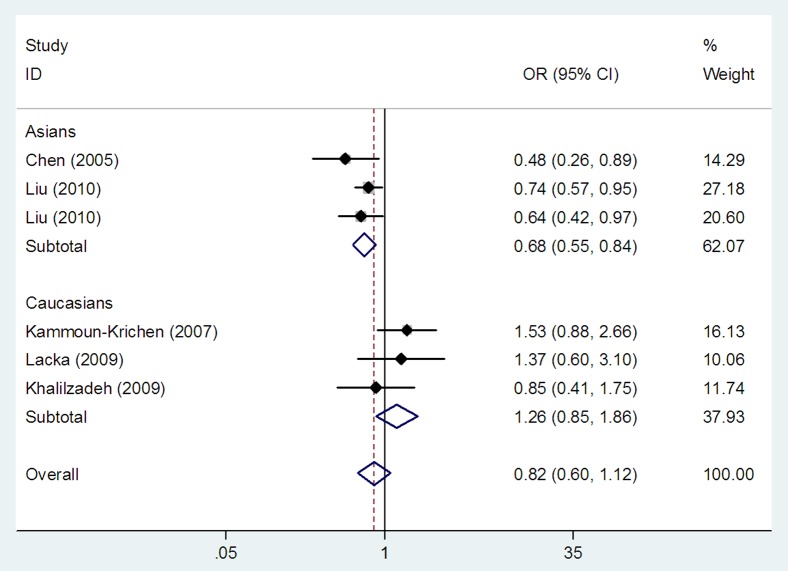
Meta-analysis with a random effects model for the association between the *IL1B* (*-511*) polymorphism and GD risk in recessive model. Each study is shown by the point estimate of the odds ratio, and a horizontal line denotes the 95% confidence interval. The pooled odds ratio is represented by a diamond. The area of the grey squares reflects the weight of the study in the meta-analysis.

Three studies investigating the *IL1B* (*-511*) polymorphism reported on Caucasian populations [22]–[24], and three on Asians [20], [25], [27]. We found an association between the *IL1B* (*-511*) polymorphism and GD risk in the overall populations in homozygote model (TT vs CC: OR = 0.86, 95% CI: 0.76–0.97, *P_h_* = 0.177, *P_z_* = 0.015) (Table 3 and Fig. 2), but not in dominant and recessive models (TT+TC vs CC: OR = 0.95, 95% CI: 0.81–1.12, *P_h_* = 0.244, *P_z_* = 0.553 and TT vs TC+CC: OR = 0.82, 95% CI: 0.60–1.12, *P_h_* = 0.052, *P_z_* = 0.205, respectively) (Table 3 and Fig. 3). In subgroup analyses stratified by ethnicity, the *IL1B* (*-511*) polymorphism was associated with GD risk in Asians in homozygote and recessive models (TT vs CC: OR = 0.81, 95% CI: 0.70–0.93, *P_h_* = 0.324, *P_z_* = 0.003 and TT vs TC+CC: OR = 0.68, 95% CI: 0.55–0.84, *P_h_* = 0.422, *P_z_*<0.001, respectively) (Table 3, Fig. 2 and Fig. 3), but not in dominant model (TT+TC vs CC: OR = 0.92, 95% CI: 0.77–1.11, *P_h_* = 0.265, *P_z_* = 0.389) (Table 3). No association between the *IL1B* (*-511*) polymorphism and GD risk was found in Caucasians in dominant model (TT+TC vs CC: OR = 1.06, 95% CI: 0.76–1.48, *P_h_* = 0.169, *P_z_* = 0.736) (Table 3), recessive model (TT vs TC+CC: OR = 1.26, 95% CI: 0.85–1.86, *P_h_* = 0.438, *P_z_* = 0.244) (Table 3 and Fig. 3) and homozygote model (TT vs CC: OR = 1.07, 95% CI: 0.82–1.41, *P_h_* = 0.405, *P_z_* = 0.600) (Table 3 and Fig. 2).

**Table 3:** Meta-analysis of polymorphisms in the *IL-1* gene and GD risk.

Polymorphism	No. of studies (Cases/Controls)	Dominant model			Recessive model			Homozygote model		
		OR (95% CI)	*P_h_*	*P_z_*	OR (95% CI)	*P_h_*	*P_z_*	OR (95% CI)	*P_h_*	*P_z_*
*IL1B (-511)*										
Total	6 (1681/1528)	0.95 (0.81–1.12)	0.244	0.553	0.82 (0.60–1.12)	0.052	0.205	0.86 (0.76–0.97)	0.177	**0.015**
Caucasians	3 (355/470)	1.06 (0.76–1.48)	0.169	0.736	1.26 (0.85–1.86)	0.438	0.244	1.07 (0.82–1.41)	0.405	0.600
Asians	3 (1326/1058)	0.92 (0.77–1.11)	0.265	0.389	0.68 (0.55–0.84)	0.422	**<0.001**	0.81 (0.70–0.93)	0.324	**0.003**
*IL1B (+3954)*										
Total	6 (972/1053)	0.63 (0.34–1.14)	<0.001	0.124	0.51 (0.17–1.55)	<0.001	0.233	0.39 (0.08–1.99)	<0.001	0.259
Caucasians	4 (406/730)	1.02 (0.95–1.09)	0.320	0.640	0.82 (0.56–1.22)	0.449	0.334	1.02 (0.60–1.72)	0.630	0.953
Asians	2 (566/323)	0.19 (0.01–5.32)	<0.001	0.328	0.04 (0.00–8.86)	0.010	0.239	0.02 (0.00–13.04)	0.003	0.240
*IL1RN (VNTR)*										
Total	6 (748/1105)	0.99 (0.80–1.22)	0.145	0.914	1.12 (0.73–1.68)	0.301	0.636	1.11 (0.73–1.70)	0.149	0.630
Caucasians	4 (516/805)	1.02 (0.81–1.29)	0.049	0.875	1.07 (0.69–1.66)	0.119	0.748	1.08 (0.69–1.69)	0.046	0.734
Asians	2 (232/300)	0.87 (0.54–1.41)	0.943	0.567	1.57 (0.34–7.15)	0.938	0.561	1.52 (0.33–6.95)	0.931	0.588

CI, confidence interval; GD, Graves' disease; IL-1, interleukin-1; OR, odds ratio; *P_h_*, *P*-value for heterogeneity; *P_z_*, *P*-value for overall effect.

* Significant associations are highlighted in bold.

### Association between the *IL1B* (*+3954*) polymorphism and GD

For this SNP, four studies were conducted in Caucasians [19], [22]–[24], while two studies were performed in Asians [20], [27]. No association between the *IL1B* (*+3954*) polymorphism and GD risk was observed in the overall populations in dominant model (TT+TC vs CC: OR = 0.63, 95% CI: 0.34–1.14, *P_h_*<0.001, *P_z_* = 0.124) (Table 3), recessive model (TT vs TC+CC: OR = 0.51, 95% CI: 0.17–1.55, *P_h_*<0.001, *P_z_* = 0.233) (Table 3) and homozygote model (TT vs CC: OR = 0.39, 95% CI: 0.08–1.99, *P_h_*<0.001, *P_z_* = 0.259) (Table 3). In subgroup analyses stratified by ethnicity, we found that the *IL1B* (*+3954*) polymorphism was not associated with GD risk in Asians in domiant model (TT+TC vs CC: OR = 0.19, 95% CI: 0.01–5.32, *P_h_*<0.001, *P_z_* = 0.328) (Table 3), recessive model (TT vs TC+CC: OR = 0.04, 95% CI: 0.00–8.86, *P_h_* = 0.010, *P_z_* = 0.239) (Table 3) and homozygote model (TT vs CC: OR = 0.02, 95% CI: 0.00–13.04, *P_h_* = 0.003, *P_z_* = 0.240)(Table 3). No association between the *IL1B* (*+3954*) polymorphism and GD risk was found in Caucasians in dominant model (TT+TC vs CC: OR = 1.02, 95% CI: 0.95–1.09, *P_h_* = 0.320, *P_z_* = 0.640) (Table 3), recessive model (TT vs TC+CC: OR = 0.82, 95% CI: 0.56–1.22, *P_h_* = 0.449, *P_z_* = 0.334) (Table 3) and homozygote model (TT vs CC: OR = 1.02, 95% CI: 0.60–1.72, *P_h_* = 0.630, *P_z_* = 0.953) (Table 3).

### Association between the *IL1RN* (*VNTR*) polymorphism and GD

Four studies evaluated this polymorphism in Caucasians [14]–[16], [22], while two studies on this polymorphism were performed in Asians [20], [21]. We found no association between the *IL1RN (VNTR)* polymorphism and GD risk in the overall populations in dominant model (22+2L vs LL: OR = 0.99, 95% CI: 0.80–1.22, *P_h_* = 0.145, *P_z_* = 0.914) (Table 3), recessive model (22 vs 2L+LL: OR = 1.12, 95% CI: 0.73–1.68, *P_h_* = 0.301, *P_z_* = 0.636) (Table 3) and homozygote model (22 vs LL: OR = 1.11, 95% CI: 0.73–1.70, *P_h_* = 0.149, *P_z_* = 0.630) (Table 3). In subgroup analyses according to ethnicity, we did not find an association between the *IL1RN* (*VNTR*) polymorphism and GD risk in Caucasians and Asians, respectively (Table 3).

### Heterogeneity and publication bias

Table 3 showed between-study heterogeneity in details. Begg's test was used to evaluate publication bias. No publication bias was present in the analysis assessing the *IL1B* (*-511*), *IL1B* (*+3954*), and *IL1RN* (*VNTR*) polymorphisms. Table 4 summarized the results of Begg's test in detail.

**Table 4:** Begg's test for evaluating publication bias.

Polymorphism	*P* for Dominant model	*P* for Recessive model	*P* for Homozygote model
*IL1B* (*-511*)			
Total	0.452	0.707	0.452
Caucasians	1.000	1.000	1.000
Asians	0.296	0.296	0.296
*IL1B* (*+3954*)			
Total	0.707	1.000	0.707
Caucasians	1.000	0.308	1.000
Asians	1.000	1.000	1.000
*IL1RN* (*VNTR*)			
Total	0.707	1.000	0.707
Caucasians	1.000	0.734	0.734
Asians	1.000	1.000	1.000

IL-1, interleukin-1.

## Discussion

Since 1995, many genetic association studies have assessed the relation of the *IL1B* (*-511*) and (*+3954*), and *IL1RN* (*VNTR*) polymorphisms with GD risk; however, the findings remain inconclusive. This may partly be owing to a small sample size of individual studies and distinct genetic background. Meta-analysis is a quantitative statistical analysis that combines the results of previous separate but related studies in order to test the pooled data for statistical significance. It has been increasingly utilized in assessing associations between genetic variants and autoimmune diseases, including multiple sclerosis (MS), type 1 diabetes, and GD [30]. In the present study, we undertook a meta-analysis of 11 published case-control studies to investigate the association between the three main *IL-1* gene polymorphisms and GD risk. The main findings were as follows: (1) the *IL1B* (*-511*) polymorphism was associated with GD risk in homozygote model in the overall populations; (2) the *IL1B* (*+3954*) and *IL1RN* (*VNTR*) polymorphisms were not associated with GD risk in any of the genetic models in the overall populations; (3) in Asians, the *IL1B* (*-511*) polymorphism was associated with GD risk in recessive and homozygote models, while the *IL1B* (*+3954*) and *IL1RN* (*VNTR*) polymorphisms were not associated with GD risk; (4) no association between the *IL1B* (*-511*), *IL1B* (*+3954*), and *IL1RN* (*VNTR*) polymorphisms and GD risk was observed in any of the genetic models in Caucasians.

IL-1B is a pluripotential proinflammatory cytokine that primarily produced by B lymphocytes, monocytes and fibroblasts. It is an important mediator for the inflammatory responses and play key roles in the triggering of immune functions, effecting nearly every cell type. IL-1B influenced thyroid cells via a number of underlying mechanisms, including down-regulation of thyroid peroxidase gene expression [31], induction of dissociation of the junctional complex [32], and inhibition of cyclic adenosine monophosphate (cAMP) and thyroglobulin production [33]. In the development of GD, infiltration of the thyroid by activated immune cells results in local release of IL-1B. It has been observed that IL-1B induces the production of IL-6, IL-8, intercellular adhesion molecule-1 (ICAM-1), and other inflammatory mediators [34]–[36]. IL-1B also enhances T cell-dependent antibody production by augmenting CD40 ligand and OX40 expression on T cells [37]. IL-1B was shown to promote differentiation of T-helper 17 (Th17) cell, the proportion of which was reported to be higher in intractable GD than that of GD in remission [38]. Therefore, *IL1B* may play a role in the pathogenesis of thyroid autoimmunity. In this meta-analysis, we observed that the *IL1B (-511)* TT genotypes were protective against GD in Asians. This SNP may affect protein expression and function [27], [39], resulting in down-regulation of inflammatory responses and resistance to develop GD. Several reasons may account for the discrepancy of results between Asians and Caucasians. First, distinct genetic background may play a role. Second, GD is a complex disease which is also related to environmental factors. Genetic susceptibility to GD may be modified by a variety of environmental exposures, leading to differences in genetic associations. Between-study heterogeneity was identified in several pooled analyses. However, when we undertook subgroup analyses according to ethnicity, heterogeneity was greatly reduced, suggesting that ethnicity was the main source of heterogeneity. Other potential factors, such as study design, sample size and genotyping methods, might also contribute to heterogeneity.

Some limitations should be acknowledged when interpreting this meta-analysis. First, our study only included publications written in English. It is possible that some relevant papers written in other languages were missed. Second, all included case-control studies were conducted in Caucasians and Asians. Therefore, the results of this meta-analysis were only applicable to these two ethnic groups. For providing additional evidence of the association between the *IL-1* gene polymorphisms and GD risk, future studies should be performed in other ethnic groups, for example in Latin Americans. Third, although we combined data from all included published case-controls assessing the *IL1B* (*-511*), *IL1B* (*+3954*) and *IL1RN (VNTR)* polymorphisms, the sample size of this meta-analysis was still relatively small, which limited the statistical power to achieve a definitive conclusion. Fourth, haplotype analysis using haplotypes constructed by these SNPs could not be conducted because relevant data provided by included studies were limited. Future association studies are needed to evaluate haplotype associations with GD risk, which will provided more insights for the effects of gene-gene interaction among these SNPs on GD risk.

In summary, our meta-analysis suggested that the *IL1B* (*-511*) polymorphism, but not the *IL1B* (*+3954*) and *IL1RN (VNTR)* polymorphisms was associated with GD risk in Asians, and there was no association between these polymorphisms and GD risk in Caucasians. Future studies should be performed using large sample numbers to establish a more definitive conclusion

## Supporting Information

Checklist S1 PRISMA Checklist.
(DOC)

Figure S1 PRISMA flow diagram.
(DOC)
