# Supplementary material for: Association between the IL1B (-511), IL1B (+3954), IL1RN (VNTR) Polymorphisms and Graves' Disease Risk: A Meta-Analysis of 11 Case-Control Studies
Source: PLoS One. 2014 Jan 21;9(1):e86077. doi: 10.1371/journal.pone.0086077 (PMC3897612; doi:10.1371/journal.pone.0086077)
Supplement: Figure S1 — PRISMA flow diagram. (DOC) [file pone.0086077.s002.doc]

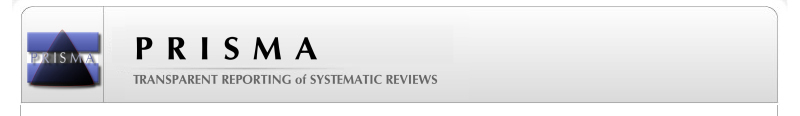
**PRISMA Flow Diagram**

**Screening**

**Included**

**Eligibility**

**Identification**

Records identified through database searching
(n = 238)

Records after duplicates and irrelevant papers were removed

(n=15)
(n = )

Full text evaluation
(n =15)

Articles were excluded due to the following reasons (n=4):

1. Duplicated sample (n=1)
2. No reported data of genotype distribution (n=2)
3. Investigation of other SNP (n=1)

Studies included in quantitative synthesis (meta-analysis)
(n = 11)
